# Investigation of anti-NMDA receptor antibodies in first episode psychosis patients

**DOI:** 10.1192/j.eurpsy.2022.929

**Published:** 2022-09-01

**Authors:** R.I. Zsigmond, L. Herman, J. Réthelyi

**Affiliations:** Semmelweis University, Department Of Psychiatry And Psychotherapy, Budapest, Hungary

**Keywords:** anti-NMDA receptor encephalitis, antibody, autoimmune encephalitis, First Episode Psychosis

## Abstract

**Introduction:**

Anti-N-methyl-D-aspartate receptor (anti-NMDAR) encephalitis is an autoimmune limbic encephalitis, where psychiatric symptoms are often the initial presentation dominant initially. These patients are mainly admitted to psychiatric wards, due to first episode psychosis (FEP).

**Objectives:**

Multiple studies analysed whether anti-NMDAR antibodies were present in the sera of schizophrenic patients, but results have not verified this hypothesis. It is possible, however, that unknown autoimmune antibodies play a role in FEP, similarly to anti-NMDAR antibodies.

**Methods:**

40 patients with FEP and 30 healthy controls have been recruited to the study. Patients with affective psychosis, drug-related psychosis and patients with diagnosed encephalitis were excluded. The sera were tested with immune fluorescent assays for anti-NMDAR antibodies. A non-specific method was used to test anti-brain antibody activity on monkey-cerebellum and rat-hippocampus slices.

**Results:**

Neither the samples from the 40 patients, nor the samples of healthy controls contained anti-NMDAR antibodies. 14 of the patients’ and only 6 of the healthy controls’ serum showed positive reaction of the neuroendothelium. These results suggest that there is a difference between the groups, although the results are not significant.

**Conclusions:**

None of the 40 patients proved positive for anti-NMDAR antibodies in agreement with previous studies. However, a higher proportion of samples from the FEP group showed activity in the neuroendothelium of non-specific immune fluorescent assays compared to healthy controls. Based on literature and on our experience, it is possible, that unknown autoimmune antibodies play role in FEP.

**Disclosure:**

No significant relationships.

